# Letter to editor about “Comparative analysis of community-acquired and hospital-acquired necrotizing soft-tissue infections in critically ill patients: a 10-year retrospective study”

**DOI:** 10.1097/JS9.0000000000003336

**Published:** 2025-09-08

**Authors:** Zhenjiang Liu, Chaolu Wang

**Affiliations:** Wangjing Hospital, China Academy of Chinese Medical Sciences, Beijing City, China

**HIGHLIGHTS**

HA-NSTIs are more likely to be polymicrobial with more enterococci; unclear pathogen interactions. Long-term monitoring of NSTIs’ microbial spectrum and drug resistance genes is needed.

HA-NSTIs have delayed antibiotic initiation; reasons (hiddenness, comorbidities, and confusion) need exploration. Antitoxin use is common in CA-NSTIs, but efficacy is unclear; more RCTs are needed.

CA- and HA-NSTIs have similar mortality, but HA-NSTIs mean longer ICU stays, possibly affecting long-term recovery and costs. More studies on long-term prognosis are needed.

The single-center, 100-case retrospective study (10 years) limits generalizability. Multicenter studies across regions/settings are needed to verify results.

*Dear Editor,*

We read with great interest the recent study by Thy *et al*, who provide a valuable comparative analysis of community-acquired (CA) and hospital-acquired (HA) necrotizing soft-tissue infections (NSTIs) in critically ill patients over a 10-year period^[1]^. This study conducted a 10-year retrospective analysis comparing CA and HA-NSTIs in 100 ICU patients, revealing distinct microbiological characteristics and management differences between the two types of infections. The research provides valuable insights for clinical decision-making in the treatment of NSTIs. However, we would like to put forward some considerations regarding the study’s design, result interpretation, and clinical application.

First, in terms of microbiological analysis, the study found that HA-NSTIs are more likely to be polymicrobial infections and have a higher prevalence of enterococci. However, the article did not elaborate on the specific interactions between different pathogens in polymicrobial infections and their impact on clinical outcomes. In addition, although the incidence of multidrug-resistant organisms (such as extended-spectrum beta-lactamase (ESBL)-producing Enterobacterales and Pseudomonas) did not differ significantly between the two groups, with the increasing problem of antibiotic resistance globally, long-term monitoring of the changes in the microbial spectrum of NSTIs is still necessary. More detailed analysis of drug resistance genes and their transmission mechanisms can help develop more targeted infection control strategies.

Second, concerning clinical management, the study pointed out that HA-NSTIs have a significant delay in antibiotic initiation. It is necessary to further explore the reasons for this delay. Is it due to the hidden nature of HA-NSTIs, the complexity of the patient’s underlying diseases, or the difficulty in distinguishing them from other HA infections? In addition, although the study found that the use of antitoxin therapy was more common in CA-NSTIs, the clinical efficacy of antitoxin therapy remains unclear due to the small sample size. More randomized controlled trials are needed to evaluate the effectiveness and safety of antitoxin therapy in different types of NSTIs.

Third, regarding the interpretation of results, the study found that there was no significant difference in mortality between CA-NSTIs and HA-NSTIs, but HA-NSTIs were associated with a longer ICU stay. This suggests that although the short-term prognosis (mortality) is similar, HA-NSTIs may have a greater impact on the patient’s long-term recovery and medical costs. Future studies can focus on the long-term prognosis of patients with NSTIs, including quality of life, functional recovery, and economic burden, to provide more comprehensive evidence for clinical decision-making.

Finally, regarding the research design, the study is a single-center retrospective study with a sample size of 100 cases. Although it covers a 10-year period, the single-center nature may limit the generalizability of the findings. NSTIs may have different epidemiological characteristics and microbial spectra in different regions and medical institutions due to variations in population structure, medical practices, and antibiotic usage patterns. For example, the low incidence of methicillin-resistant *Staphylococcus aureus* (MRSA) reported in the study may be related to the local epidemiological situation in France, but this may not be applicable to regions with a high MRSA prevalence. Therefore, multicenter studies involving different geographical regions and medical settings are needed to verify these results.

In summary, this study provides important insights into the differences between CA-NSTIs and HA-NSTIs. We believe that expanding the sample size, conducting multicenter studies, and conducting in-depth research on the mechanisms and long-term prognosis of the disease will further improve our understanding and management of NSTIs. We appreciate the authors’ contributions to this field and look forward to more in-depth studies to guide clinical practice.

## Data Availability

Not applicable.

## Statement

The article was written in accordance with the TITAN Guidelines 2025^[2]^.
